# Feasibility of single position laparoscopic radical nephrectomy and tumor thrombectomy for left renal cell carcinoma with high-risk Mayo grade 0 and 1 tumor thrombus

**DOI:** 10.1186/s12894-021-00924-2

**Published:** 2021-12-22

**Authors:** Adili Keranmu, Mingshuai Wang, Yajian Li, Feiya Yang, Wasilijiang Wahafu, Dong Chen, Jing Liang, Kaopeng Guan, Nianzeng Xing

**Affiliations:** 1 State Key Laboratory of Molecular Oncology, National Cancer Center/National Clinical Research Center for Cancer/Cancer Hospital, Chinese Academy of Medical Sciences and Peking Union Medical College, Beijing, 100021 People’s Republic of China; 2 Present Address: Department of Urology, National Cancer Center/National Clinical Research Center for Cancer/Cancer Hospital, Chinese Academy of Medical Sciences and Peking Union Medical College, No. 17, Panjiayuan South Li, Chaoyang District, Beijing, 100021 People’s Republic of China; 3 Department of Urology, Institute of Urology, Beijing Chaoyang Hospital, Capital Medical University, Beijing, 100020 People’s Republic of China; 4 Department of Pathology, National Cancer Center/National Clinical Research Center for Cancer/Cancer Hospital, Chinese Academy of Medical Sciences and Peking Union Medical College, Beijing, 100021 People’s Republic of China

**Keywords:** Renal cell carcinoma, Laparoscopy, Tumor thrombus, Nephrectomy

## Abstract

**Background:**

To explore the feasibility of single-position laparoscopic radical nephrectomy (LRN) and tumor thrombectomy for left renal cell carcinoma with high-risk Mayo 0 and 1 tumor thrombus (TT).

**Methods:**

All patients with left renal cell carcinoma and venous TT (high-risk Mayo grade 0 and 1) who were performed single-position LRN and tumor thrombectomy were involved. After the renal artery was controlled by Hem-o-lok, the left renal vein was dissected through descending colon mesentery. The left renal vein was divided by EndoGIA for high-risk Mayo grade 0 TT. For Mayo grade 1 TT, part of the inferior vena cava was blocked by a bulldog clamp after milking the TT into the left renal vein and the inferior vena cava was sutured after complete excision of the TT.

**Results:**

3 patients were involved and operations were performed successfully without conversion to open surgery. The mean operation time was 136 min and the mean estimated blood loss was 60 mL. No postoperative complications occurred.

**Conclusions:**

It is feasible to control left renal vein and partial inferior vena cava through descending colon mesentery in a single position during LRN and tumor thrombectomy for the treatment of high-risk Mayo grade 0 and 1 TT.

## Introduction

Invasion of the venous system is one of the clinical features of locally advanced kidney cancer. Approximately 4–10% of locally advanced renal cell carcinomas have venous tumor thrombus (TT) [1]. Studies have shown that radical nephrectomy and inferior vena cava TT removal for patients with locally advanced renal cancer can effectively improve the prognosis, and the 5-year tumor-specific survival rate is 40–65% [2]. In recent years, robot-assisted/laparoscopic surgery has become the first choice for the treatment of kidney cancer with venous TT for minimal invasion. For patients with renal cell carcinoma and Mayo grade 0–2 TT, minimally invasive surgery can achieve long-term tumor control similar to open surgery and has better perioperative outcomes [3]. Left renal vein TT is more complicated than right renal vein TT and the length of Mayo grade 0 TT determines the complexity of the surgery. If it is a low-risk Mayo grade 0 TT, the end of the TT is on the left side of the mesenteric artery, the left renal vein can be fully controlled by traditional approach. If it is a high-risk Mayo grade 0 TT or even grade 1 TT, traditionally two steps were utilized including management of the inferior vena cava and removement of the TT, and then nephrectomy after changing the surgical position. In order to simplify the procedure, we explored the trans-mesocolon approach to fully dissect the left renal vein to the inferior vena cava in single position to treat high-risk Mayo 0-grade and grade 1 TT.

The aim of the study was to explore the feasibility of single position transperitoneal laparoscopic radical nephrectomy (LRN) and high-risk Mayo 0 and 1 tumor thrombectomy and the surgical techniques were described in detail.

## Methods

### General information

All patients with left renal cell carcinoma and venous TT (Mayo grade 0–1) who were performed single-position LRN and tumor thrombectomy were involved. The 3 enrolled patients were males, aged 54–57 years old, with a median age of 57 years old. Urinary tract ultrasound, enhanced MRI, MRA, chest CT and bone scan were performed before surgery to determine the location, size and metastasis of the tumor. The clinicopathological data are outlined in Table 1.

**Table 1:** The patient’s clinicopathological characteristics

Case	Age (year)/sex	BMI (kg/m^2^),	ECOG score standard	ASA grades	Tumor thrombus level	Other organs with metastasis	Kidney puncture	Preoperative treatment
1	57/M	20.18	1	II	Mayo 1	Multiple lung metastases	Renal cell-derived carcinoma	Targeted therapy + immunotherapy
2	54/M	25.95	1	I	Mayo 1	Left adrenal gland metastasis	Renal cell carcinoma	Targeted therapy
3	57/M	22.53	1	II	Mayo 0	Multiple lung metastases + Retroperitoneal lymphadenopathy	Renal clear cell carcinoma	Targeted therapy + immunotherapy

*M* male, *BMI* body mass index, *ECOG* Eastern Cooperative Oncology Group, *ASA* the American Society of Anesthesiologists

Case 1 imaging examination revealed a left kidney tumor with venous TT (Mayo stage 1) and multiple lung metastases. The kidney biopsy confirmed clear renal cell carcinoma with poorly differentiated and accompanied by necrosis. Two cycles of axitinib combined with PD-1 inhibitor were provided for neoadjuvant treatment.

The imaging examinations of case 2 showed a left kidney tumor with venous TT (Mayo grade 1), accompanied by left adrenal gland metastasis. The pathology from kidney biopsy demonstrated clear renal cell carcinoma and the patient received 2 cycles of axitinib for neoadjuvant treatment.

The imaging examination of case 3 showed left kidney tumor with retroperitoneal lymphadenopathy, left renal vein TT (Mayo grade 0) and multiple lung metastases. The kidney biopsy showed clear renal clear cell carcinoma. The TT shrunk to high-risk Mayo grade 0 after 3 months neoadjuvant treatment of sintilimab and axitinib.

### Position and trocar placement

**Fig. 1:**
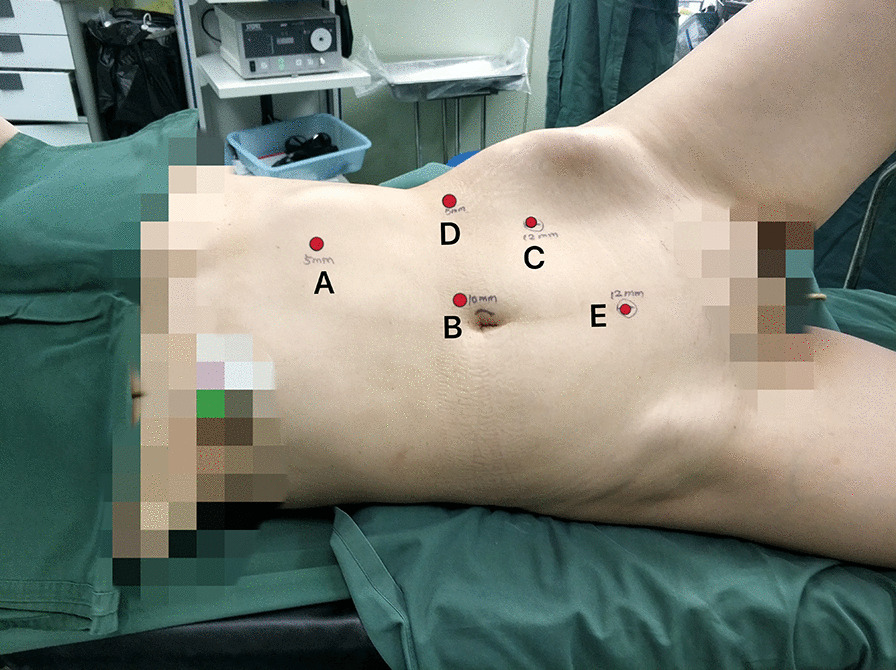
The position and trocar placement

After successful anesthesia, the patient was placed in a supine position obliquely 70° on the right side, fixed with a lumbar pillow, and raised the lumbar bridge. The position of the trocar was shown in Fig. 1: The 5 mm trocar A, located 1 cm below the costal margin of the left clavicle midline, was used to puncture into the abdominal cavity and then the pneumoperitoneum was established. The pressure is maintained at 12 mmHg. The 10 mm trocar B was used for laparoscopic lens, locating on the outer edge of the affected rectus abdominis muscle. The 12 mm trocar C is placed at the midpoint of the connection between the left anterior superior iliac spine and the belly button. The 5 mm trocar D is located at the flat umbilicus of the anterior axillary line. The 12 mm trocar E is established at the midpoint of the connection between the umbilical cord and the pubic symphysis.

### Techniques of nephrectomy and tumor thrombectomy

*The operation is performed according to the principle of treating the artery first and then treating the vein. The specific method is as follows* after the left descending colon and part of the sigmoid colon lateral ligament were incised, the small intestine was pulled to the mid-abdomen. It’s worth noting that to avoid the loss of mesangial blood vessels during dissociation. A dense connective tissue can be seen in the middle of the dorsal medial side of the kidney, and the arterial pulsation can be seen under it. After open the connective tissue and the left renal vein could be seen. At the same time, the adrenal vein and reproductive vein are disconnected when separating the renal vein. The laparoscopic interventional ultrasound was used to detect the end of the TT, then the left renal vein was divided by EndoGIA for Mayo grade 0 TT and part of the inferior vena cava was blocked by a bulldog clamp after milking the thrombus into renal vein for Mayo grade 1 TT. After completely remove the TT, the inferior vena cava was sutured and finally radical nephrectomy was performed. It should be noted that when handling the inferior vena cava, we usually clamp the root of the left renal vein with a bulldog clamp. We only clamp a part of the wall of the inferior vena cava, which will not affect the return of the inferior vena cava and lumbar vein. Therefore, there is no need to deliberately isolate the lumbar vein and cut it off.

## Results

All operations were successfully completed without transfer to open surgery, and no postoperative complications occurred. The mean operation time was 136 min (45–196) min and the mean estimated blood loss was 60 ml (30–100) mL. The normal diet was restored on postoperative two.

No complications such as fever, intestinal obstruction, intestinal fistula, and urine leakage occurred. The wound recovered well when the patient was discharged from the hospital, and there was no wound infection. There were no complications such as bleeding and pulmonary embolism during the perioperative period.

**Fig. 2:**
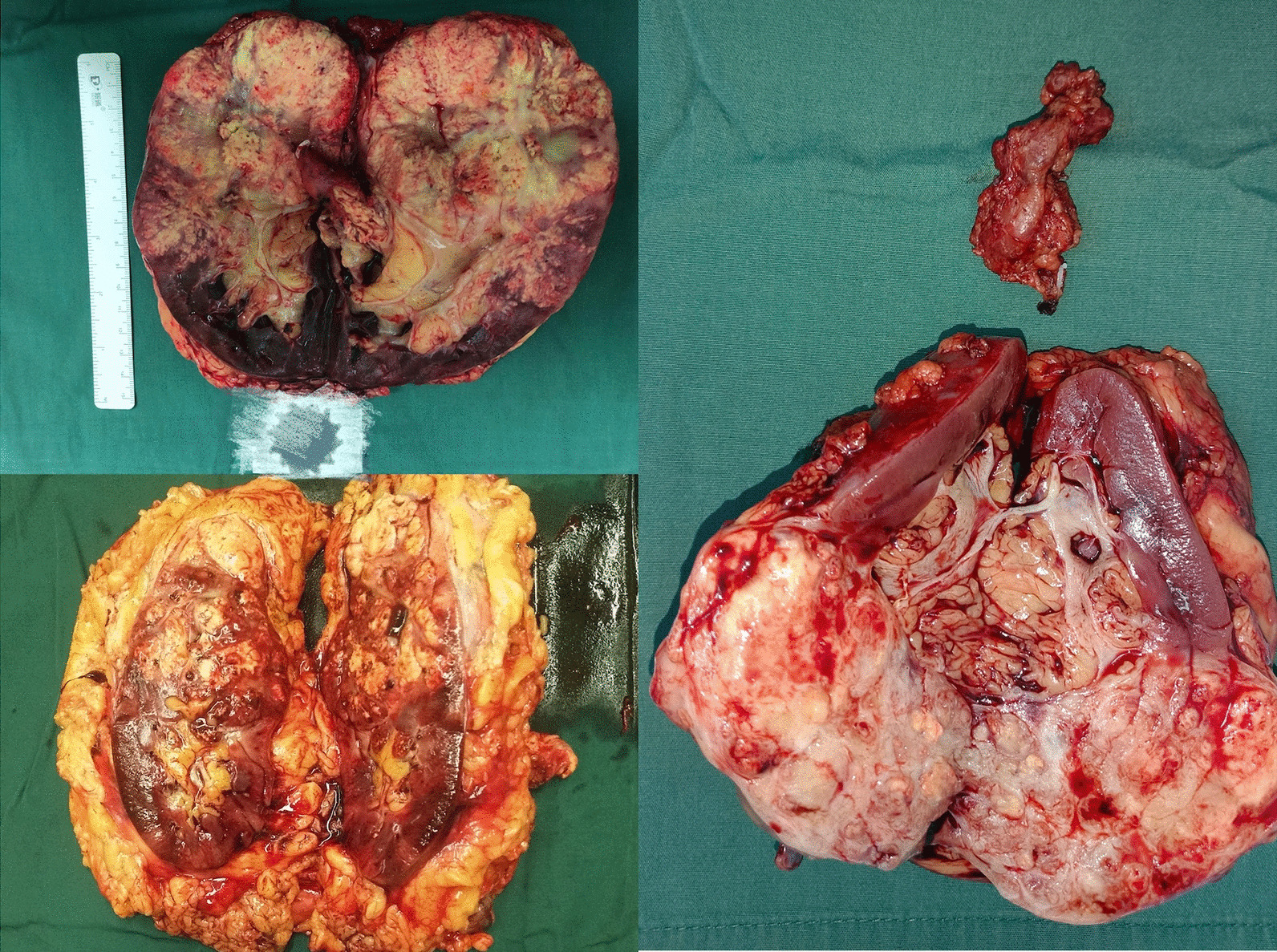
Postoperative pathological gross specimen

**Fig. 3:**
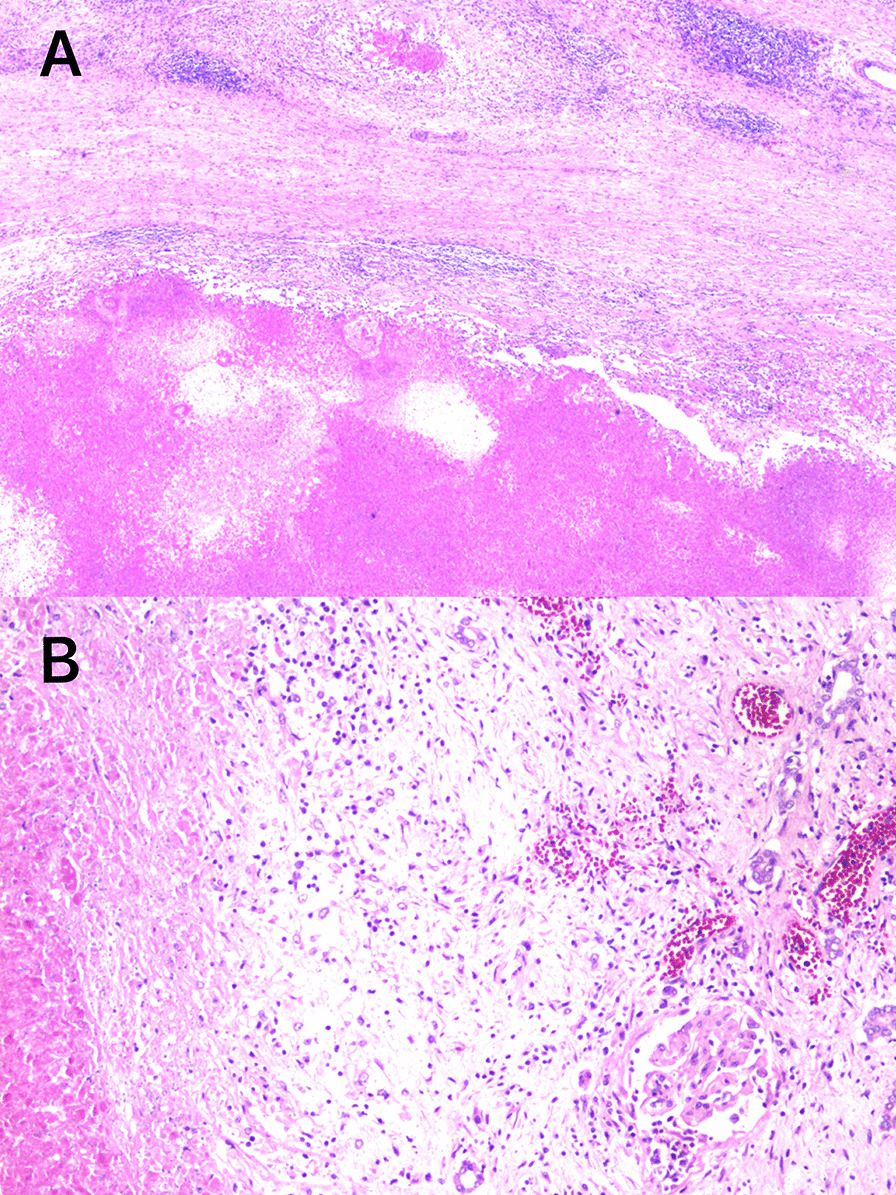
Microphotograph of renal cell cancer (hematoxylin and eosin stain, **A** magnification × 4, **B** magnification × 10)

The postoperative pathology of two cases showed the tumors were clear renal cell carcinoma (postoperative pathological gross specimen was shown in Fig. 2). The postoperative specimen of another patient showed large necrotic foci, and no residual tumor was seen under the microscope. Combined with the medical history, it was consistent with pathological complete remission (pCR) (Fig. 3). The median follow-up period of 3 patients was 14 months, and there was no tumor recurrence or metastasis (Table 2).

**Table 2:** The patient’s perioperative clinical data

Case	Operation time	Intraoperative blood loss	Postoperative drainage volume	Drainage tube placement time (days)	Catheter placement time (days)	Postoperative hospital stay (days)	Major complications in 90 days
1	166	100	5421	4	1	4	None
2	196	50	82	2	1	4	None
3	45	30	2760	12	1	11	None

## Discussion

Invasion of the venous system is one of the clinical features of locally advanced renal cancer, including invasion of the renal vein and inferior vena cava. The aim of the study was exploring the feasibility and safety of single-position LRN and tumor thrombectomy for left renal cell carcinoma with high-risk Mayo 0 and 1 TT. In short, the operation in our study was completed in a single position without changing the position. The surgical procedures were reasonable and the techniques could be quickly mastered, saving anesthesia and operation time. The surgical outcomes were encouraging including small trauma, quick postoperative recovery, short hospital stay, and few postoperative complications.

For the left renal cell carcinoma with high-risk Mayo 0 and 1 TT, the traditional robot-assisted/laparoscopic surgical method is to remove the affected kidney, then change the position and remove the TT. Another method is performing embolization of the left renal artery before the operation and treating the inferior vena cava and TT during the operation time, then change the position to remove the left kidney. Using traditional method increases the risk of surgery such as damaging the mesangial blood vessels. At the same time, anesthesia and operation time are prolonged. Open surgery, the standard approach for treating inferior vena cava thrombus, is technically complex, has high complication rates, and requires multidisciplinary cooperation [4]. Furthermore, these methods are failed to fully reflect the advantages of minimally invasive surgery. In recent years, scholars at home and abroad have explored single-position LRN and tumor thrombectomy for left renal cell carcinoma with high-risk Mayo 0 and 1 TT, but there are few related reports [5]. In our study, the operation was completed in a single position and a single way without changing the position. The surgical procedures were reasonable and standardized.

In 2009, our team took the lead in completing and reporting retroperitoneal LRN and tumor thrombectomy and summing up experience. In 2012, we completed the first complete LRN and grade II vena cava tumor thrombectomy in China. In view of the fact that the transperitoneal route has a greater impact on the gastrointestinal tract and the patient’s recovery is slow after surgery, in 2014, the retroperitoneal route of radical nephrectomy plus the removal of grade II inferior vena cava TT was completed for the first time in the world [6]. Based on years of attempts, our center has accumulated rich experience in renal cancer surgery with venous tumor thrombi. Therefore, we are more confident to explore single-position LRN and tumor thrombectomy for left renal cell carcinoma with high-risk Mayo 0 and 1 TT.

The key points and techniques of this study are as follows: 1. Reasonably design the patient’s position and scientifically place the Trocar position to facilitate the operation and cooperation of the surgeon and assistant. The patient’s position and Trocar position are the key to the success or failure of a single-position operation; 2. The method of finding and processing the renal pedicles using laparoscopic procedures: For the left renal pedicle, the kidney can be pushed to the ventral side with a vascular clamp. A dense connective tissue can be seen in the middle of the dorsal medial side of the kidney, and arterial pulses can be seen below to open the connective tissue. The left renal artery can be seen [7]. After the renal artery is found, the hem-o-lok clamp is used to block the renal blood flow, but not cut off. After the renal vein is processed, then cut off the renal artery in order to reduce blood oozing; 3. Determine the corresponding surgical strategy according to the height of the TT; 4. Using the laparoscopic ultrasonography in this operation to detect the location of the TT in the vein, determine the size of the TT, and detect the specific position of the end of the TT to determine how to block the vein [8–10]; 5. Strictly follow the principle of tumor free and sterile; 6. Before the specimen is taken out, suture the peritoneum of the incised paracolonic sulcus with an absorbable thread to Reduce the risk of intestinal adhesions and intestinal obstruction which is conducive to the recovery of intestinal function; 7. The specimens must be taken out in the specimen bag, which can reduce the risk of tumor implantation and the length of the incision. Female patients can use the NOSES procedure to remove the specimens from the vagina, which can achieve less trauma, fast recovery, good cosmetic effects, and short hospital stay and many other advantages.

The height of the TT determines the specific treatment strategy, especially the surgical strategy, so a grading system based on the height of the TT came into being. Mayo Clinic’s five-level classification of tumor thrombi has been widely used in clinical work [1]. The 3 cases in this study are high-risk Mayo 0 and 1 TT patients. Based on conventional knowledge and traditional diagnosis and treatment methods, for grade 0 or I tumor thrombosis, and TT that hardly extends to the inferior vena cava, the thrombus can be removed. Squeeze the renal vein back, and then place it in the renal vein orifice with a vascular clip to isolate it to avoid complete blockage of the inferior vena cava [11]. For patients with left renal tumor with venous tumor thrombosis, we have confirmed through this study that it can be done under single position laparoscopy.

In laparoscopic renal vein/inferior vena cava TT removal surgery, because we cannot accurately determine the position of the end of the TT with the naked eye, it is very dangerous to blindly test before blocking the renal vein or inferior vena cava which increasing the risk of TT shedding. As mentioned earlier, laparoscopic ultrasonography is used in this operation to detect the location of the TT in the vein, determine the size of the TT, and detect the specific position of the end of the TT to determine how to block the vein [8–10]. After the inferior vena cava is incised and the thrombus is removed, check again to prevent residual tumor thrombi. It can prevent the TT from remaining or falling off due to insufficient blocking range, and it can also avoid damage to the inferior vena cava due to excessive dissociation, making the operation more accurate and greatly reducing the risk of surgery [9, 10, 12]. Therefore, hospitals that perform such operations recommend routine preparations for Laparoscopic ultrasonography before surgery [10].

The use of neoadjuvant systemic therapy to reduce locally advanced tumors to promote surgical resection and improve survival is used in various malignant tumors [13]. The rapid development and approval of anti-angiogenesis targeted therapy for metastatic renal cell carcinoma since 2006 has aroused people’s interest in the potential application of these drugs in reducing tumor thrombosis levels [14]. For example, in a study by Cost, the median height of venous TT decreased by 1.5 cm, and one patient was successfully reduced from Mayo Clinic grade 4 to grade 3 [15]. All 3 patients in this study received targeted and immunotherapy before surgery. One patient received Sintilimab and axitinib for 3 months and then Venous TT was reduced from Mayo 1 to high grade 0. Another patient received axitinib combined with PD-1 treatment for 2 cycles and Postoperative pathology suggests pCR. However, the results of these studies are diverse. It has been reported that 5–44% of cases have reduced TT height, 28–91% of cases have stable disease, and 5–28% of cases have advanced tumor thrombi [16–18]. So far, there is no first-level evidence to support the use of targeted drugs before venous TT resection.

In summary, compared with traditional surgical methods, the single-position LRN and tumor thrombectomy for left renal cell carcinoma with high-risk Mayo 0 and 1 TT through abdominal cavity has the advantages like standardized methods, simple steps, less trauma, less bleeding, and quick postoperative recovery. It is a safe and effective minimally invasive technique for the treatment of renal cell carcinoma with venous TT. We initially discussed the safety and feasibility of this surgical method. However, due to the small number of clinical samples, patients and operations involved are summarized by a single center, its technology promotion, clinical efficacy and prognostic evaluation need to be further verified by multi-center, large sample size and long-term follow-up. Therefore, it is recommended to carry out a comparative study with classic laparoscopic procedures to explore the advantages of different procedures.

## Data Availability

The datasets used and/or analyzed during the current study are available from the corresponding author on reasonable request.

## Abbreviations

LRN: Laparoscopic radical nephrectomy
TT: Tumor thrombus
MRI: Magnetic resonance imaging
MRA: Magnetic resonance angiography
CT: Computed tomography
BMI: Body mass index
pCR: Pathologic complete response
IVC: Inferior vena cava
